# Otoscopic and tympanometric findings in infants with cleft lip and palate

**DOI:** 10.1016/S1808-8694(15)31096-X

**Published:** 2015-10-19

**Authors:** Mariza Ribeiro Feniman, Adriana Guerta de Souza, José Carlos Jorge, José Roberto Pereira Lauris

**Affiliations:** 1 Associate Professor - Department of Speech and Hearing Therapy - Dentistry School of Bauru - University of São Paulo (USP); 2 Speech and Hearing Therapist - Department of Speech and Hearing Therapy - Dentistry School of Bauru - University of São Paulo (USP). Student at the Professionalizing Practices in Audiology - FOB/USP; 3 MS. in human communications disorders - Hospital de Reabilitação de Anomalias Craniofaciais da Universidade de São Paulo, HRAC/USP, ENT physician - HRAC/USP. Assistant Professor - Department of Speech and Hearing Therapy - Dentistry School of Bauru - University of São Paulo (USP); 4 Associate Professor - Department of odontopediatrics, orthodontics and collective health - Dentistry School of Bauru (FOB) - University of São Paulo (USP). Universidade de São Paulo - Faculdade de Odontologia de Bauru - Departamento de Fonoaudiologia

**Keywords:** cleft palate, infant, otoscopy, acoustic impedance tests

## Abstract

Tympanometry plays a fundamental role in the identification of middle ear alterations, which are frequent in the population with cleft lip and palate.

**Aim:**

do a retrospective analysis of the otoscopy and tympanometric exams of infants with cleft lip and palate who were not operated. Retrospective study.

**Materials and Methods:**

we analyzed 273 charts from infants with cleft lip and palate whom, from March 1996 to April of 2002 underwent pneumatic otoscopy and tympanometry with a 226 Hz probe.

**Results:**

We did not find statistical significance in the otoscopic and tympanometric findings considering ears and genders. We observed 84% of alterations in otoscopy (opacification/83.4%, visible fluid in the middle ear /1.5%, the ear drum does not move during inflation /1.8 and retraction/0.7) and 65% in tympanometric curves (B/38%), A/36.5%, As/21%, C/4% and Ad/0.5%).

**Conclusion:**

female and male infants with cleft lip and palate did not differ as far as otoscopic and tympanometry findings are concerned. All types of tympanometric curves were present, and types A and B were the most frequent ones. Ear drum opacification was the most frequent otoscopic finding. Pneumatic otoscopy identified a larger number of alterations when compared to conventional tympanometry.

## INTRODUCTION

Studies have reported that children with craniofacial anomalies, especially those with cleft lip and palate, have a high incidence of middle ear alterations.^1^, ^2^, ^3^, ^4^, ^5^

During decades, tympanometry has been a broadly accepted method to assess middle ear function^6^, as a fast, safe, non-invasive and easy to use procedure.

Considering the fundamental role played by tympanometry in the identification of middle ear alterations, which have a high incidence in the population with cleft lip and palate, we thought it was necessary to carry out a retrospective study of tympanometric findings in infants with this congenital malformation in order to help characterize the person's audiologic profile.

The goal of this study is to carry out a retrospective analysis of the results of otoscopic and tympanometric exams in infants with cleft lip and palate that were not operated.

## MATERIALS AND METHODS

After proper approval by the Ethics in Research Committee (protocol # 140/2005UEPCEP), we carried out a retrospective study of 300 medical charts from infants with a unilateral trans-foramen cleft in the left incisive tooth^7^, chosen randomly, which tympanometric and otoscopy evaluations were carried out from March, 1996 to April of 2002. All the infants included in this study did not present any genetic syndrome associated, nor had been submitted to any repair surgery either for malformation and/or otological. This study was carried out in 2005.

From the medical charts we studied data associated with gender, surgical condition, results from tympanometric and otoscopic exams and patient's age at the time of the exams.

For the tympanometry, we used the Grason Stadler Middle Ear Analyzer version 2 impedanciometer. The immittance tone frequency was of 226Hz (conventional). The tympanometric measures were automatically carried out by the equipment at a speed of 50 deca-Pascals per second (daPa/s). The type of tympanometric curve followed the classification proposed.^8^

For the pneumatic otoscopic exams, we used the Heine otoscope (Diagnostik-Otoskope K 100).

The exams (tympanometric and otoscopic) were carried out one day before the lip surgery.

The otoscopic findings were classified in: without alteration, when through the otoscopy we saw an intact tympanic membrane (intact, translucid and mobile during inflation); and with alteration, when there was fluid in the middle ear, opacification, retraction and immobility of tympanic membrane during inflation.

Tympanometric curves were classified in normal and altered. It was normal when the A-curve was obtained, and altered for the other types found (B, C, As and Ad).

The data obtained was organized in Tables to facilitate analysis and presentation. We used the chi-squared statistical treatment for the data. The significance level adopted was 1% (p < 0.01).

## RESULTS

The random choice of medical charts showed that 27 infants had already been submitted to some surgical procedure and were taken off the study, thus making up a study group of 273 infants (546 ears), being that 161 (59%) were males and 112 (41%) were females.

The age of the infants at the time of the exams varied between 3 and 5 months.

Table 1, Table 2 showed the distribution of otoscopic findings and tympanometric curves, according to gender and ear, respectively. Table 3 shows the distribution and percentage of classification of such findings.

**Table 1:** Otoscopic findings according to gender and ear.

	Male		Female		
Otoscopic findings	RE	LE	RE	LE	Total
Tympanic membrane opacification	131	130	93	93	447
Tympanic membrane opacification, not mobile during inflation and visible fluid	2	2	1	0	5
Opacification, tympanic membrane retraction, static during inflation	1	1	0	0	2
Tympanic membrane opacification and retraction	0	0	1	1	2
Static during inflation and visible fluid	1	1	0	1	3
No alteration	26	27	17	17	87
Total	161	161	112	112	546

**Table 2:** Tympanometric curve frequency distribution according to gender and ears.

	Male		Female		
Tympanometric curves	RE	LE	RE	LE	Total
B	61	65	46	37	209
A	67	58	33	41	199
As	27	30	29	28	114
C	6	6	4	5	21
Ad	0	2	0	1	3
Total	161	161	112	112	546

**Table 3:** Frequency and percentage distribution of tympanometric curves and otoscopic findings.

	Otoscopic findings		Tympanometric curves	
	N (ears)	%	N (ears)	%
With alteration	459	84	347	65
Without alteration	87	16	199	35
Total	546	100	546	100

The statistical study did not show significant statistical difference between the genders (p=0.13763) and ears (p=0.58783) for otoscopic findings, as well as for tympanometric curves (p=0.45534) and (p=0.52375), respectively.

## DISCUSSION

The results from this study show that tympanic membrane opacification, with or without other alterations, had the higher incidence (83.4%) in the population with cleft lip and palate that we studied. Such otoscopic finding was also noticed in other papers published in the literature.^3^^,^^5^^,^^9^^,^^10^ Usually, its presence is the result of a thickness in the tympanic membrane, the presence of effusion, or both.^11^

Melker and Harris et al. studied and support the usefulness of pneumatic otoscopy as a low cost and adequate diagnostic tool able to predict the presence of fluid (effusion) in the middle ear, and ear drum position^12^ and mobility are considered the most important diagnostic indicators.^12^, ^13^, ^14^, ^15^ Takahashi et al. report that, in general, when the middle ear is filled with air, the ear drum moves in response to pressure produced by the pneumatic otoscopy in the external acoustic meatus, and it does not move when there is fluid present there.^16^ When the ear drum does not move during inflation, with or without visible fluid, opacification and ear drum retraction, was present in 1.8% of the ears in the population sampled for this study.

In eighty-seven ears (16%) of this present study, no alteration was determined by otoscopy, which can reflect lack of middle ear effusion.

In regards of tympanometric exams, 38% (209 ears) of type-B tympanometric curves were identified by tympanometry with a low frequency probe in the present study with infants from three to five months of age with cleft lip and palate and who were not operated. This type of tympanogram is a strong evidence of otitis media with effusion^13^ and is strongly associated with age, characterized by a high occurrence in the first six months of life.^17^ Younger children have a higher risk of developing otitis media with effusion, because the Eustachian Tube is in a more horizontal position when compared to older children, which makes it difficult to drain this fluid in the middle ear.^18^ Paradise and Bluestone, studied 138 infants (zero to 20 months of age) with cleft palate, showing that all had otitis media in their first three months of life.^19^ Broen et al. noticed normal middle ear function in few children with this non-repaired congenital abnormality.^20^

Andrews et al. also identified a type-B curve in 83% of 40 infants with 3 months of age who had cleft palate, however using tympanograms obtained from a high frequency probe.^21^

However, type-A tympanograms, indicating normal middle ear function were also found in 36.5% of the population sampled in the present investigation. An incidence of 64.1% of type-A tympanometric curve was reported by Namyslowski and Kubik in a study carried out with 85 children with cleft palate, using tympanometry with the 226HZ probe.^22^ We stress that studies by Balkany et al., Hunter and Margolis and Baldwin have reported normal tympanograms obtained with the 226Hz probe, in the confirmed setting of middle ear pathology.^23^, ^24^, ^25^ Abnormal tympanometric results, using multifrequency evaluation, were obtained in ears that previously had the type A tympanometric pattern with low frequency probe.^13^^,^^22^

Type As is a curve that presents a reduced maximum compliance peak, seen in middle ears with some fluid or with ossicle fixation, which partially reduces mobility^26^, present in 21% of the population sampled in this study.

Seen in only 4% of the infants in our sample, the type-C tympanometric curve, not usually found in patients with cleft lip and palate^3^, show a highly negative pressure in the middle ear, and may indicate a transition from a normal ear and an ear filled with fluid.^27^

Harris et al. proved that the types As and C tympanogram curves may be associated with normal middle ear function, as well as the presence of fluid. They also reported that some children with normal low frequency tympanograms had abnormal results found by the multifrequency probe, resulting from monomeric tympanic membranes or mechanical disorders in the middle ear.^13^

A low incidence (0.5%) of the type Ad tympanometric curve was seen in our study. In the literature surveyed, this type of alteration was not reported in infants with cleft palate and lip.

Looking at Table 3, one can see a similar behavior for otoscopic findings and tympanometric curves, thus indicating an increase in alterations when compared to the normal cases. However, a greater percentage of altered otoscopic findings (84%) was identified by the pneumatic otoscopy when compared to those with alterations in tympanometric curves (65%) obtained by tympanometry with the 226Hz probe. When we studied this higher frequency of alterations in the pneumatic otoscopy, one considered that the otoscopy-tympanometry relation is not very uniform for children below six months of life.^28^ Moreover, in the present investigation, there may have been some false negative results associated with the tympanograms, because a recent study^25^ reports tympanometries not being proper when used in low frequency in infants before 5 months of age, because it can produce wrong results.^21^^,^^25^ Tympanometries with high frequency probe is stressed, because it is better able to discriminate ears with and without otitis media with effusion in infants^29^^,^^30^ and provide more detailed information on the middle ear acoustic and mechanical status.^13^

It is important to highlight that for the tympanometric findings obtained in this study, we used conventional tympanometry, in other words, a low frequency probe (226HZ). Thus, it is highly important to continue this study by making a comparative analysis using the multifrequency probe for tympanometry, to help characterize the audiologic profile of the population sampled, thus contributing to identify the most effective method to be used in the auditory evaluation of infants with cleft lip and palate in their first years of life.

## CONCLUSION

Male and female infants with cleft lip and palate were not different in their tympanometric curves, or in their otoscopic findings. All types of tympanometric curves were present, and type A was the most frequently found. Tympanic membrane opacification was the most frequent otoscopic finding. Pneumatic otoscopy identified a greater number of alterations when compared to conventional tympanometry.

## References

[1] Feniman MR, Donadon DR, Vieira JM. (1999). Audição de pacientes com fissura isolada de lábio e com fissura de palato: um estudo comparativo. J Bras Fonoaudiol.

[2] Kemaloglu YK, Kobayashi T, Nakajima T. (1999). Analysis of the craniofacial skeleton in cleft children with otitis media with effusion. Int J Pediatr Otorhinolaryngol.

[3] Handzic-Cuk J, Cuk V, Gluhinin M, Risavi R, Stajner-Katusic S. (2001). Tympanometric findings in cleft palate patients: influence of age and cleft type. J Laryngol Otol.

[4] Sheahan P, Miller I, Sheahan JN, Earley MJ, Blayney AW. (2003). Incidence and outcome of middle ear disease in cleft lip and/or cleft palate. Int J Pediatr Otorhinolaryngol.

[5] Ramana YV, Nanda V, Biswas G, Chittoria R, Ghosh S, Sharma RK. (2005). Audiological profile in older children adolescents with unrepaired cleft palate. Cleft Palate J.

[6] Ferekidis E. (2003). The use of advanced tympanometry techniques in the differential diagnosis of middle ear pathology. Ent News.

[7] Spina V, Psillakis JM, Lapa FS, Ferreira MC. (1972). Classification of cleft lip and cleft palate. Suggested changes. Rev Hosp Clin Fac Med São Paulo.

[8] Jerger J. (1970). Clinical experience with impedance audiometry. Arch Otolaryngol.

[9] Feniman MR, Souza-Freitas JÁ. (1991). Achados otoscópicos e audiométricos em portadores de fissura pós-forame incisivo. Acta Awho.

[10] Fernandes DR, Feniman MR, Piazentin-Penna SHA. (2001). Avaliação da função da orelha média pela timpanometria em crianças com fissura de lábio e palato antes das restaurações das lesões labiais e palatinas. J Bras Fonoaudiol.

[11] Bluestone CD, Klein JO. (1995). Otitis media in infants and children.

[12] Melker RA. (1993). Evalution of the diagnostic value of pneumatic otoscopy in primary care using the results of tympanometry as a reference standard. Br J Gen Pract..

[13] Harris PK, Hutchinson KM, Moravec J. (2005). The use of tympanometry and pneumatic otoscopy for predicting middle ear disease. Am J Audiol.

[14] Rees GL, Freeland AP. (1992). The effects of anesthesia on tympanograms of children undergoing grommet insertion. Clin Otolaryngol Allied Sci.

[15] Rodriguez WJ, Schwartz RH, Thorn MM. (1995). Increasing incidence of penicillin- and ampicillin-resistant middle ear pathogens. Pediatr Infect Dis J.

[16] Takahashi H, Honjo I, Hasebe S, Sudo M, Tanabe M. (1999). The diagnostic and prognostic value of eardrum mobility in otitis media with effusion. Eur Arch Otorhinolaryngol.

[17] Engel J, Anteunis L, Chenault M. (2000). Otoscopic findings in relation to tympanometry during infancy. Eur Arch Otorhinolaryngol.

[18] Zeisel SA, Roberts JE. (2003). Otitis media in young children with disabilities. Infant Young Child.

[19] Paradise JL, Bluestone CD. (1974). Early treatment of the universal otitis media of infants with cleft palate. Pediatrics.

[20] Broen PA, Moller KT, Carlstrom J, Doyle SS, Devers M, Keenan KM. (1996). Comparison of the hearing histories of children with and without cleft palate. Cleft Palate Craniofac J.

[21] Andrews PJ, Chorbachi R, Sirimanna T, Sommerlad B, Hartley BEJ. (2004). Evaluation of hearing thresholds in 3-month-old children with a cleft palate: the basis for a selective policy for ventilation tube insertion at time of palate repair. Clin Otolaryngol.

[22] Namyslowski G, Kubik P. (1996). 226HZ and high frequency tympanometry in children with cleft lip and/or palate. Otolaryngol Pol.

[23] Balkany TJ, Berman SA, Simmons MA, Jafek BW. (1978). Middle ear effusion in neonates. Laryngoscope.

[24] Hunter LL, Margolis RH. (1992). Multifrequency tympanometry: current clinical application. Am J Audiol.

[25] Baldwin M. (2006). Choice of probe tone and classification of trace patterns in tympanometry undertaken in early infancy. Int Aud.

[26] Pensak M., Tami TA. (1998).

[27] Combs JT. (1994). The diagnosis of otitis media: new techniques. Pediatr Infect Dis J.

[28] Smith CG, Paradise JL, Sabo DL, Rockette HE, Kurs-Lasky M, Bernard BS (2006). Tympanometric findings and probability of middle-ear effusion in 3686 infants and young children. Pediatrics.

[29] Kei J, Allison-Levick J, Dockray J, Harrys R, Kirkegard C, Wong J (2003). High-frequency (1000Hz) tympanometry in normal neonates. J Am Acad Audiol.

[30] Margolis RH, Bass-Ringdahl S, Hanks WD, Holte L, Zapala DA. (2003). Tympanometry in Newborn Infants-1 kHz Norms. J Am Acad Audiol.

